# What do you need to know when preparing to give a talk at an international dermatology conference? Insights and practical recommendations

**DOI:** 10.1097/JW9.0000000000000184

**Published:** 2024-11-14

**Authors:** Hemali Shah, Rose Parisi, Luísa Polo Silveira, Roni P. Dodiuk-Gad

**Affiliations:** a Department of Dermatology, University of Pittsburgh Medical Center, Pittsburgh, Pennsylvania; b Department of Medicine, Albany Medical Center, Albany, New York; c School of Public Health, Columbia University, New York, New York; d Department of Dermatology, Emek Medical Center, Afula, Israel; e Division of Dermatology, Department of Medicine, University of Toronto, Toronto, ON, Canada; f Department of Dermatology, Bruce Rappaport Faculty of Medicine, Technion Institute of Technology, Haifa, Israel

**Keywords:** conference, dermatology, networking, presentation, public speaking

## Abstract

**Objectives:**

To provide a checklist for presentation preparation at dermatology conferences, discuss important factors to consider when preparing a presentation, and recommend strategies for effective presentations and networking.

**Data Sources:**

With a combination of personal experience and literature review of PubMed database and dermatology society resources, this article serves as the first comprehensive guide for how to prepare a talk for an international dermatology conference.

**Conclusion:**

Conferences are an excellent opportunity to learn more about yourself, your field, and others throughout the world. Well-prepared presentations have the potential to greatly impact your audience and expand your connections. The authors provide a step-by-step discussion and checklist that thoroughly addresses the logistics, operations, scientific content, and social aspects that are important to know when preparing to give a presentation in the field of dermatology.

**What is known about this subject in regard to women and their families?**

- In the literature, women have reported significantly greater fear than men while talking to authority figures, giving a talk in front of an audience, being the center of attention, speaking up at a meeting, and giving a report to a group.

**What is new from this article as messages for women and their families?**

- Our article addresses this discrepancy by serving as a guide for women in dermatology on how to effectively prepare to give a talk at a conference.

## Introduction

Public speaking is the most commonly reported fear in the general population.^1^ Public speaking anxiety is a subtype of social anxiety disorder defined by the Diagnostic and Statistical Manual of Mental Disorders that is prevalent in 15 to 30% of the general population.^2^ Social anxiety disorders are the most common type of anxiety disorder, with a lifetime prevalence of 12.1%.^3^ Sixty-four percent of undergraduate students have reported experiencing public speaking anxiety.^2,4^ With such a high prevalence of public speaking anxiety among university and graduate students, the questions arise: what are the implications of this in future and current physicians and how do we help counter this fear? The answer, in part, lies in practice and preparation.^5^ Upon review, we found that there is currently no literature regarding how to effectively prepare a talk within the field of dermatology. In an effort to develop this literature, our authors share insights and recommendations.

### Anecdote

During the World Congress of Dermatology in 2023, I was invited to give a 5-minute talk at the International Alliance for Global Health Dermatology World Congress Scientific Meeting.^6^ It was my first time speaking at a medical conference; starting at the World Congress of Dermatology was overwhelming for me. To prepare myself, I did my homework: I memorized my speech, prepared my slides in advance, included animations, and practiced numerous times in front of the mirror and with my mentor via Zoom. In my mind, I was ready, and I believed that nothing could go wrong. However, after the first 2 minutes of my speech, I could barely feel my feet. My legs were trembling, my voice was failing, and my mouth was dry. By the end of the 5 minutes, I felt mentally and physically exhausted. Even though I had received all the necessary support from my mentors and from International Alliance for Global Health Dermatology, it was still very challenging for me. Contrary to what I felt, many people praised me for my presentation. What I perceived as obvious, my shaking hands and my failing voice, went unnoticed. For them, the message I was conveying was much more important than those minor details. This experience changed my perspective. I realized how critical we can be of ourselves compared to how others perceive us. Furthermore, I started to think about what I could have done differently to boost my confidence and improve my overall presentation.^6^

There is a paucity of comprehensive step-by-step guides that address the logistics, operations, scientific content, and social aspects of preparing to give a talk during dermatology conferences.^7,8^ With a combination of personal experience and literature review, this article is intended to provide medical students, dermatology trainees, and practicing dermatologists with a comprehensive guide on how to prepare for conference presentations.

## Presentation preparation guide

Delivering a scientific presentation is a way of disseminating observations, hypotheses, research findings, and their implications to your peers. Such presentations have the power to leave lasting impressions on both presenters and their audiences, yet they can be quite challenging to execute effectively.^8^ The general framework when crafting a presentation is to provide an overview of your topic, provide an explicit message, and then summarize take-home points concisely.^9^ While this may sound intuitive, actionably doing this proves difficult in many situations.

The initial step is crafting your presentation. An exceptional presentation is one that is concise and communicates with clarity. This article primarily focuses on developing a podium presentation with insights and strategies that can also be applied to other contexts, such as poster displays.

Alexandrov and Hennerici^7^ introduced the PRESENT model for preparation, which we will incorporate into our approach. This model outlines a systematic framework with several key components: one should be prepared to plan from the start (place integral parts of the presentation in a logical sequence), reduce the amount of text and visual aids to the bare minimum, elucidate the methods, summarize results and key messages, effectively deliver, note shortcomings, and transform one’s own and others’ current thinking.^8^

### Prior to the presentation

Before preparing your slides, it is essential to verify the requirements outlined by the conference organizing committee, such as specific slide backgrounds. It is common for conferences to provide a standardized slide template for you to use. In cases where such a template is not provided, it is advised to incorporate the conference logo discreetly in the corner of your slides to provide context for your presentation.

When planning the sequence of a presentation, consider including the following: a title slide that precisely mirrors the submission’s title as it appears in all conference documents (eg, website, program), a list of authors with their current affiliations, and the conference name. Additionally, if you have any potential conflicts of interest, it is essential to disclose these early in your presentation, as well as any off-label drug usage. For presentations focused on original research, it is most effective to adhere to the format outlined in your abstract, typically following the IMRaD (Introduction, Methods, Results, and Discussion/Conclusions) framework.^8^

### Presentation development

When formatting presentation slides, it is important to visualize your presentation from the perspective of the audience. Whether you are preparing a poster or slides, short statements and large font sizes are preferred. The text on a poster should be legible from a distance of 6 feet and slides should be easily readable from the back of an auditorium. Therefore, it is crucial to use fewer words and to minimize the use of small graphics.^8^ Only essential information should be provided in the clearest manner. Colors with straightforward contrast (eg, black text on a white background) should be used to avoid overwhelming or confusing the audience. Avoid fading elements in and out on your slides to ensure that all text remains consistently clear for your readers. In addition, always include references on your slides, whether the information is from published sources or not. If it is information from your own unpublished manuscript, you may say so.

If using patient photographs, make sure to maintain anonymity by covering patient eyes and ensure you have written and signed patient consent.

To avoid overcrowding your slides, it is important to view them as prompts to guide your spoken content rather than as a primary text.^8^ Organize your presentation from the framework of “What is my take-home message for the audience?” or “What do I want people to remember from this talk?.” The most time and space should be allotted to the results section of your presentation as this is the point of your talk. The most thought and consideration should be allotted to your discussion and conclusion, as these are the key take-home messages for your audience.^8^

The most important step of presentation preparation involves rehearsal to ensure a clear and precise message for each slide. Particularly in the early stages of one’s career, it is beneficial to practice in front of friends, family members, and mentors, remaining receptive to constructive feedback. If possible, have your mentors or colleagues ask questions to help you prepare to address any questions that may arise when you present. The presentation should genuinely reflect your personality and effectively convey your narrative.

### Resources available

There are a few resources available online to help in preparing a research project and presentation. For example, the American Academy of Dermatology recommends using the company Language Scientific as the preferred resource for translating medical and pharmaceutical information between over 150 languages.^10^ For non-native English speakers, you may want to consult with an English speaker regarding your pronunciation and grammar. Preferred Reporting Items for Systematic Reviews Health has research protocol development and abstract writing resources that may be helpful during the preparation stage.^11^ Standards for Quality Improvement Reporting Excellence published online guidelines that outline how to effectively organize an abstract and article, which can also help guide the organization of a presentation.^12^ These guidelines are intended to create more transparency and structure for authors who may be planning to organize an abstract for conference submission.

## Pre-conference preparation

### Scientific level

From a scientific standpoint, conference preparation involves a thorough understanding of the project to be presented, encompassing background research, methodology, data, limitations, and conclusions. It is critical to verify the accuracy of the presentation with the latest literature, regardless of when it was initially written. It is also essential to acknowledge the extent of your knowledge to answer questions appropriately. Being honest by admitting a lack of knowledge when a question is asked is never inappropriate.

It is also helpful to adopt a reviewer’s perspective about your research to effectively prepare for questions. This involves considering other articles related to your research and discerning the distinctions between your work and others in the field. Furthermore, it is also crucial to be well-prepared by understanding your audience, particularly the experts attending your session who specialize in your topic. This entails familiarizing yourself with the conference chairs, moderators, and fellow speakers, along with their relevant publications, to anticipate potential inquiries.

### Operational level

On an operational level, it is important to be aware of the allocated time for your presentation, including whether it includes the questions and answers (Q&A) section. On average, you should expect to allocate around 1 minute per slide when planning your presentation.^8^ However, it is worth noting that this timing may vary from slide to slide, so practice sessions should include timing yourself.

Additional essential considerations involve ensuring your presentation aligns with the conference’s formatting requirements and opting for a portable document format (PDF) as your final document type to maintain formatting integrity rather than using PowerPoint. It is equally important to know who will be introducing you and the information they may require, including your curriculum vitae (either a concise or complete version), a photo, your current educational standing (eg, medical student, resident, attending, etc.), and your affiliation(s). Make sure you are aware of the specific room name and number where you are presenting and the scheduled time of your presentation. It is also important to be aware of the timing for uploading your slides in the speakers’ room before your talk.

Before your presentation, it is helpful to visit the presentation room and assess the room layout, microphone height and placement, and accessibility to the presenter’s screen. It is prudent to have multiple backup versions of your presentation on a flash drive, in your email, and on another cloud storage in both PDF and PowerPoint formats in case of technical issues.

### Social level

When presenting at a scientific conference, your aim extends beyond sharing your scientific work. Conferences also present an opportunity to make yourself visible within a community of interest, facilitate networking, and foster potential collaborations. Therefore, it is essential to be prepared for networking endeavors. In advance, create straightforward business cards with your name, affiliation, and contact details for distribution. You can include a QR code linked to your curriculum vitae or the title of your presentation. Before the conference, consider inviting people you know to attend your talk, or when distributing your business card at a conference event, let them know when and where you are presenting.

Of note, if you are presenting internationally, consideration of cultural differences and societal norms is essential. For international conferences, it can be a kind gesture to greet your audience and to conclude your remarks in the audience’s native language. Personalizing your presentation by thanking the conference organizers/chairs or person who invited you (if applicable), acknowledging notable people in the field, extending appreciation to colleagues, and incorporating images from your home or from the presentation location can enhance your engagement with the audience. Including your email address and, if comfortable, your phone number at the end of your presentation is optional. Leveraging technology, such as a QR code linked to your LinkedIn profile and WhatsApp contact information, facilitates postpresentation networking and contact. Before the conference, assess the attendees in your session and identify those with whom you aim to connect, considering potential opportunities.

In terms of conference attire, business casual is usually appropriate, though it is never wrong to dress business formal. For your presentation, consider wearing layers to ensure you are neither too hot nor too cold. For men, ties are not necessary, but blazers are recommended. As with any professional situation, ensure your clothes appear professional and make sure you are wearing comfortable shoes that you can walk and stand in for long periods of time.

As previously discussed, public speaking anxiety is highly prevalent and can affect any individual, regardless of education or experience level. While this is a common issue many people face, it should not prevent you from enjoying the experience of presenting. Although it can be intimidating, we encourage all students, residents, and practicing physicians to jump into presenting as early as possible, whether it is in small or large audiences. At the beginning, all people feel uncomfortable, but with practice, this will feel more natural.

### Logistical level

From a logistical standpoint, it is prudent to schedule your arrival at least 24 hours before the meeting to mitigate the risk of flight delays or other travel-related issues. It is also helpful to visit the room in which you are presenting and ensure that your slides are compatible with the technology 24 hours before your presentation, allowing ample time for troubleshooting if necessary.

To minimize the risk of travel delays, try not to schedule your arrival on the same day as your presentation. When booking accommodations, opt for a hotel close to the conference venue. While conference-provided accommodations are often the most convenient option, if you opt for alternative accommodation, ensure you are well-acquainted with the location, directions, and local travel options.

In cases of international travel, it is essential to be aware of the following key points: is an official letter from the conference organizers required to enter the country; is a travel visa necessary in advance; and have there been any changes in conference dates and times on the meeting’s official website? In addition, ensure that all contact information you provide to the conference organizers is updated for any potential communication needs, and have a point-of-contact’s information readily available for yourself. A checklist summarizing this information is presented in Table 1.

**Table 1:** A dermatology conference presenter’s checklist

Before the conference
Communication with conference organizers: Verify the requirements outlined by the conference organizing committee, such as specific slide backgrounds or headers, presentation length, disclosures, etc. Make sure to have a PDF final version. Send your curriculum vitae (according to the guidelines), your professional photo, your current educational standing, affiliations, and contact information (email, phone number) to the conference organizers. Be aware of the allotted time for your talk. Check whether it encompasses the questions and answers part.
Preparation of your talk: Practice your talk with regard to time adherence and let your mentors and colleagues hear your talk, advise you, and also ask you questions. For non-native English speakers, you may want to consult with an English speaker regarding your pronunciation and grammar. In the preparation of your talk, take into consideration the research work conducted by other speakers and chairs of the session, if relevant. On the last slide of your talk, consider adding your email, phone number, and QR code of your LinkedIn or other professional social media you are active in. Have multiple backup versions of your presentation on a flash drive, in your email, and on another other cloud storage in both PDF and PowerPoint formats.
Preparation for social networking: Create straightforward business cards with your name, affiliation, and contact details for distribution. Consider inviting relevant colleagues who are planning to attend the meeting to join your talk.
International travel: In cases of international travel, it is essential to be aware of the following: whether you require an official letter from the conference organizers to enter the country, confirm if a travel visa is necessary in advance, and double check all dates and times on the meeting’s official website, which is typically more up to date than your invitation. Book your flight always at least 24 hours before the day of your talk.
At the conference before the talk
Getting comfortable in the presentation room: Before your presentation, visit the presentation room, stand at the podium, and assess the room layout.
Technology assessment: Put your slides on the presentation screen and make sure the visual format is correct. Check the slide clicker, microphone height, placement, functionality, and sound quality, as well as the accessibility to the presenter’s screen. If your presentation includes multimedia content like videos or audio, test them.
Enjoy the experience of giving a talk!

PDF, portable document format.

## At-conference preparation

### Tasks on arrival

Upon arriving at the conference venue, complete your check-in with the conference team. Then, proceed to the speakers’ room to upload your presentation slides in PDF format. If you can, seek permission from the conference staff to visit the room where your presentation will be held. At this point, it is important to advocate for yourself, as seeing the presentation room beforehand can alleviate potential anxiety in an already stressful situation. When you enter the presentation room, approach the stage and look out at the audience. Determine your preferred seating position in relation to the stage so you know where you will be walking from. Note the placement of the presenter’s screen to ensure it allows you to monitor your slides without constantly looking behind you. Ensure it does not obstruct your view of the audience. Check the microphone height and test its mobility and sound quality. If possible, display your presentation on the screen and check the clicker or buttons to make sure you are comfortable advancing the slides. If your presentation includes multimedia content like videos or audio, test them in advance. It is generally discouraged to use a laser pointer, as it can be distracting or malfunctioning; instead, consider highlighting points directly on the slide itself using different colors, outlines, or shapes.

### Presentation time

Prior to the presentation, it is important to address any personal needs, including using the restroom. Studies have shown that the concept of “power posing,” or standing in a position of confidence, such as with your chest upright and hands on your hips before an important interview or event enhances performance.^13^ This is a tool that may be used in private to reduce stress and increase confidence before a presentation. When you are called to the stage, be sure to have water within reach at the podium. Approach the stage at an unhurried pace—there is no rush to start your presentation. Upon reaching the podium, take a moment to compose yourself. Pause, count to 3, breathe deeply, and then begin your presentation, rather than starting immediately upon reaching the stage. Be mindful of your body language, as it can significantly influence the impact of a presentation.^14^ Position yourself well in relation to the microphone and recognize where your hands are. Maintaining an engaged stance with your hands open and gently resting on the podium might be a good option, showing the audience you are receptive to questions.

During your presentation, it is essential to address the entire audience by periodically scanning the room. Establish eye contact with a few audience members to foster engagement. Understand that if you observe people using their phones, it should not be taken personally, or negatively impact your presentation. Familiarize yourself with the timer’s location, whether it is on the podium or managed by an individual in the audience who will signal when your allocated time is running out. Avoid relying solely on a wristwatch, as you do not want to be frequently glancing downward. Be aware of how the timer is set and whether it counts forward or backward to effectively manage your presentation timing.

After your presentation, when it is time for the Q&A session, be prepared to respond to questions. From an etiquette perspective, never engage in arguments with individuals posing questions. Regardless of the nature of the inquiry, always express gratitude for their participation. Maintaining professionalism, politeness, and humility is paramount. Recognize that you may not possess answers to every question and know that it is entirely appropriate to respond with a courteous acknowledgment such as, “Thank you for that interesting question. I do not know the answer at this moment, but I will undertake further research and get back to you.” In situations where an individual provides inaccurate information, avoid confronting them or telling them they are wrong. Instead, respond respectfully by sharing the correct information as you understand it, without explicitly refuting their point.

### Networking

An often overlooked aspect of conferences is the importance of utilizing your mentors and networking effectively. The question of how to network effectively is highly individualized. To shed light on this topic, our authors will share their personal experiences to provide insights.

There are many examples of how to network. Some examples include enjoying meals with colleagues in between talks or in the evenings, whether this is at formal conference events, pharmaceutical representative dinners, or personally organized meals. Try to meet with mutual colleagues from different cities and countries, whether this is to merge connections or to simply catch up with one another. When you meet a new connection, get their contact information and send them a message right away. It is helpful to take a photo with them and send that with your name and affiliation to provide a memory cue for yourself and that individual.

It is also important to learn from advancements and strategies in other dermatology departments. Try to also network with pharmaceutical representatives. Try to make time to attend social events or alumni reunions. It is important to reconnect with fellow students, residents, or attendings who you have worked with in the past in-person or remotely. If you have additional free time, you may also want to reserve some time to explore the city the conference is in as well as try the local cuisine.

## Postconference tasks

Following the conference, you will most likely be exhausted. Conferences can be not only mentally draining due to information overload but also emotionally demanding from the effort invested in forging professional connections. Use your time to recover and to solidify these new relationships. Taking this opportunity to extend gratitude to the conference organizers for their hospitality and for the opportunity to present might be a good idea. You may also consider reaching out to individuals you are interested in collaborating with in the future.

## Conclusion

Conferences are an excellent opportunity to learn more about yourself, your field, and others throughout the world. Well-prepared presentations have the potential to greatly impact your audience and expand your connections. However, there are multiple often overlooked areas to consider in preparation before a conference from scientific to social levels that we have outlined above to provide an effective checklist for students, residents, and delegates in dermatology to follow when planning to give a talk at a conference.

## Conflicts of interest

The authors made the following disclosures: R.P.D.-G. consults for Janssen, Sanofi, AbbVie, Novartis, Pfizer, La Roche-Posay, Dexcel, Devintec Pharma, and Eli Lilly. RP, HS, and LPS have no relevant conflicts of interest to disclose.

## Funding

None.

## Study approval

N/A

## Author contributions

Conceptualization, literature search, data analysis, writing—review and editing, supervision: All authors. Writing—original draft preparation: HS, RP, LPS. All authors have read and agreed to the published version of the manuscript.
